# Contrasting effects of experiencing temporally heterogeneous light availability versus homogenous shading on plant subsequent responses to light conditions

**DOI:** 10.1186/s12870-023-04229-4

**Published:** 2023-05-03

**Authors:** Deng Wang, Shu Wang, Li-Xia Li, Ye-She Wang, Ke-Nian Ling-Hu, Jia-Xing Chen

**Affiliations:** 1 College of Urban and Rural Construction, Shaoyang University, Shaoyang, 422000 China; 2 College of Forestry, Forest Ecology Research Center, Guizhou University, Guiyang, 550025 China

**Keywords:** Biochemical response, Constant shading, Fluctuating light conditions, Karst species, Late growth, Morphological response, Photosynthetic physiological traits

## Abstract

Temporally heterogeneous environments is hypothesized to correlate with greater plasticity of plants, which has rarely been supported by direct evidence. To address this issue, we subjected three species from different ranges of habitats to a first round of alternating full light and heavy shading (temporally heterogeneous light experience), constant moderate shading and full light conditions (temporally homogeneous light experiences, control) and a second round of light-gradient treatments. We measured plant performance in a series of morphological, biomass, physiological and biochemical traits at the end of each round. Compared to constant full light experience, temporally heterogeneous light conditions induced immediate active biochemical responses (in the first round) with improved late growth in biomass (during the second round); constant moderate shading experience increased photosynthetic physiological and biomass performances of plants in early response, and decreased their late growth in biomass. The karst endemic species of *Kmeria septentrionalis* showed greater improvement in late growth of biomass and lower decrease in biochemical performance, due to early heterogeneous experience, compared to the non-karst species of *Lithocarpus glaber* and the karst adaptable species of *Celtis sinensis*. Results suggested plants will prefer to produce morphological and physiological responses that are less reversible and more costly in the face of more reliable environmental cues at early stage in spite of decreased future growth potential, but to produce immediate biochemical responses for higher late growth potential when early environmental cues are less reliable, to avoid the loss of high costs and low profits. Typical karst species should be more able to benefit from early temporally heterogeneous experience, due to long-term adaptation to karst habitats of high environmental heterogeneity and low resource availability.

**Supplementary Information:**

The online version contains supplementary material available at 10.1186/s12870-023-04229-4.

## Background

Plants are able to cope with the environmental heterogeneity at both spatial and temporal scales through phenotypic plasticity, defined as the ability of a genotype to produce different phenotypes in different environmental conditions [1, 2]. Greater plasticity is hypothesized to correlate with high heterogeneity in resource availability [3-6]. Many studies have been focused on plant plasticity in relation to spatial-scale environmental heterogeneity [7-9], much less attention has been paid to the relationship between time-scale environmental heterogeneity and plasticity. However, temporally heterogeneous environments may be more ubiquitous than environmental heterogeneity at spatial scale, plants can experience fluctuations in light, water and nutrient availability at scales of hours, days and months in their lifetime It is reported that *Convolvulus chilensis* experiencing the greatest temporal variation in nature has shown the greatest plasticity in four traits [10]. There is a correlation between greater interannual variation in precipitation and higher plasticity in *Senna candolleana* [11]. However, plasticity may not necessarily increase with higher spatial and temporal heterogeneity in nutrient availability [12]. Inconsistent conclusions have revealed the lack of direct evidence for effects of environmental heterogeneity on plant plasticity [13].

Light availability is crucial for plant survival and growth, especially at seedling and juvenile stages and it can be highly heterogeneous at both spatial and temporal scales. For example, in Guiyang in southwest of China, cloudy and rainy days can often last for one or two weeks continuously, before sunny days recur, especially in winter. Generally, fluctuation or temporal heterogeneity in light availability should be more common than spatially heterogeneous light conditions, but little is known about whether and how such fluctuation can affect plant later performance and response. Studies have reported that early experience with either inundation or drought, or alternate inundation and drought, can improve plant late performance in different water conditions [14, 15]. It suggests early experience with alternate full light and shading can also have beneficial effects. However, it is reported that shade-induced response at early stage constrains plant later response to shade [16], suggesting the otherwise. The shading treatment during early temporally heterogeneous light experience may limit the growth potential of plants in later shade, contrary to the hypothesis for correlation between greater plasticity and environmental heterogeneity [3, 4, 17]. It is also possible that low light availability differs from water stress, as the lack or excess of water supply reduces plant growth immediately, while shade can induce rapid and active responses in plants, such as the extra stem elongation [18-20]. Generally, deficiency in resources per se may trigger some mechanisms for improving later performance and survival, at the same time incurring associated costs. Consequently, experience with temporally heterogeneous resource availability may have complex effects on plant later growth, depending on environmental factors and/or species, which we know little about. Here we adopted three species from different habitat ranges and measured a large number of traits in different aspects, to explore this issue and behind mechanisms.

Karst is a highly special geomorphology formed by the natural processes of solution and leaching of soluble rocks, generally carbonate rocks [21]. Karst ecosystems are characterized by great complicacy, highly spatio-temporal heterogeneity in abiotic factors such as light, water, and nutrient availability [22-24]. Species from karst habitats are more likely to experience spatial and temporal heterogeneity in resource availability than those from other habitats, thus may have developed stronger ability to deal with highly heterogeneous environments. Although there are a lot of studies on responses of karst species to abiotic factors such as drought, nutrients, and calcium [25-28], information is extremely scarce for the correlation between plasticity and heterogeneous environments in these species.

To investigate whether and how previous experience with temporal heterogeneity in light availability will alter plant later response to light conditions, we conducted a greenhouse experiment with three species from different habitat ranges, including a karst-endemic species of *Kmeria septentrionalis* (grows in karst habitats only), a karst-adaptable species of *Celtis sinensis* (grows in both karst and normal habitats) and a non-karst species of *Lithocarpus glaber* (grows in normal habitats only). We subjected these plants to an initial round of alternating full light and shading treatments (temporally heterogeneous light conditions), with constant full light and moderate shading (two temporally homogeneous treatments) as control (considered as early experience), before another round of light-gradient treatments to test plant plastic responses (late response), and measured a number of morphological, biomass, physiological and biochemical traits for each individual plant at the end of either round. We asked the following questions: (1) compared to early constant full light or shade experience, does previous exposure to temporal heterogeneity in light availability improve plant subsequent performance or response to shading in different sets of traits? and (2) do such effects differ between different species or between different late conditions?

## Materials and methods

### Study materials

We used three arbor species of *Kmeria septentrionalis* (Magnoliaceae), *Lithocarpus glaber* (Fagaceae) and *Celtis sinensis* (Ulmaceae), representing karst endemic species, karst adaptable species and non-karst species. They are all heliophytes, and frequently occur in different ranges of natural habitats of Guizhou. *K. septentrionalis* is an evergreen species that grows to a height of up to 18 m (breast diameter of up to 40 cm), flowering between May and June, with dioecious and unisexual flowers, and fruiting between October and November. It has strong tolerance for drought and low nutrient availability, and only distributes in karst limestone regions of northern Guangxi and southeastern Guizhou in China. *C. sinensis* is a deciduous species with a height of up to 20 m. It is a dioecious and monoecious species, flowers between April and May, and fruits between September and November. It can adapt to a wide range of soil conditions from slightly acidic or alkaline, neutral to calcareous soil, and is ubiquitous in roadside, hillside and forest edge of both karst and non-karst regions. *L. glaber* is an evergreen species with a height of up to 15 m (breast diameter of up to 40 cm). It is a monoecious species. It tends to grow in thick and loose soil of high fertility, and generally distributes in non-karst broad-leaved forests to the south of Qinling Mountains of China.

Seeds of *K. septentrionalis* were collected in autumn of 2018 from Maolan National Natural Reserve in Libo County of Guizhou Province and cultivated to seedlings in local plantations. Seeds of *C. sinensis* and *L. glaber* were collected between autumn and winter in 2018, from natural wild populations in southeast of Guiyang of Guizhou Province. They were pretreated with corresponding methods and stored at 5 °C.

### Experimental design and treatments

The experiment was conducted in a greenhouse on West Campus of Guizhou University in Guiyang (106° 42’ E, 26° 34’ N; altitude ~ 1020 m). The region has a typical subtropical monsoon climate, with annual average temperature of 15.3 °C, relative humidity of 77%, total precipitation of 1129.5 mm, and insolation duration of 1148.3 h. Seeds were grown in trays in the middle of March, 2019 (12/12 hours of light/dark conditions, temperature of 25 ± 3 °C and humidity of 60% ± 2%). At the stage of three to four leaves, seedlings were transplanted into pots (22 cm in diameter, 20 cm in depth), filled with disinfected and sterilized limestone soil. The initial size (the basal diameter of stem) for each individual plant was measured before light treatments were initiated, when seedlings had grown for about 30 days .

**Fig. 1:**
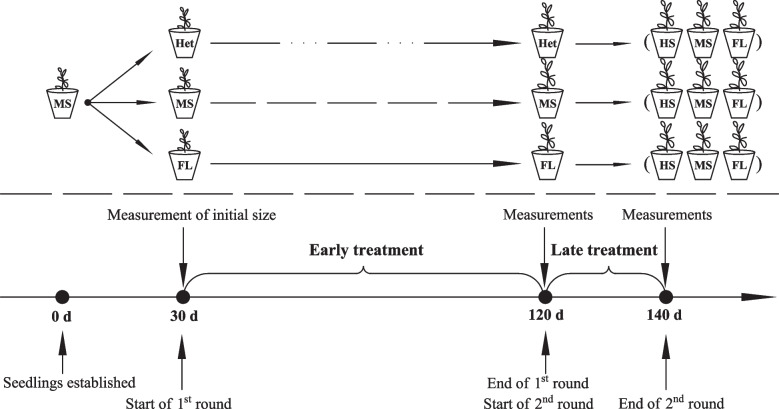
Experimental design showing the two rounds of treatments with timing in this study. The 1st round of treatments included early temporally heterogeneous light (Het, alternating full light and heavy shade), homogeneous moderate shading (MS, CK_1_) and full light (FL, CK_2_) conditions. The 2nd round of treatments included heavy shading (HS), moderate shading (MS) and full light (FL)

A split-plot experimental design was implemented, with the first round of treatments (early experience) as the main factor, and the second round of treatments (for testing late responses) and species as sub-factors. The early treatments included temporally heterogeneous light conditions (E_het_), temporally homogeneous moderate shading (E_hom−MS_) and full light (E_hom−FL_); the late treatments included full light (L_FL_), moderate shading (L_MS_) and heavy shading (L_HS_; Fig. 1). Seedlings of three species were randomly distributed within each light treatment. The temporally heterogeneous light treatment was set up by alternating full light and heavy shading treatments for three cycles. Plants were first subjected to full light conditions for 15 days, then transferred to heavy shading for another 15 days, before entering another cycle of treatments. The early treatments lasted for 90 days in total, before a subgroup of individuals for each species in each treatment was sampled to measure plant performance in early experience. All the other plants entered the second round of treatments, including full light (L_FL_), moderate shading (L_MS_), and heavy shading (L_HS_) conditions, which lasted for another 20 days, before all individuals were harvested and measured. Due to the enormous workload (three species and twelve kinds of light treatments in total), and similar sizes of seedlings for the same species, we reduced the amount of samples to nine replicates per species per treatment, to make the experimental system easier to control. Therefore, for each species in each of early treatments (the first round), there were a total of 36 individual plants at the beginning, 9 individuals were sampled for measurements at the end of the first round, the remaining 27 individuals entered one of three late treatments (the second round) and measured at the end of experiment, making a total of 9 replicates × 3 species × 3 experiences + 9 replicates × 3 species × 3 experiences × 3 late conditions = 324 samples. The entire experiment lasted for about five monthst.

The heavy shading and moderate shading treatments were set up by covering the experimental plot with a black nylon mesh with light transmittance of 35% and 70% respectively, full light treatment was established by covering a transparent sheet over the plot to ensure the levels of other abiotic factors such as wind, temperature approach to the two shading treatments as much as possible. The average values of light intensity for full light, moderate shading and heavy shading treatments were 1010.02 ± 22.05, 675.67 ± 11.17, and 332.33 ± 8.09 µmol·m^− 2^·s^− 1^ respectively inside the greenhouse in June, 2019, equivalent to 90%, 60%, and 30% of natural full light, with 1147.33 ± 45.67 µmol·m^− 2^·s^− 1^ outside the greenhouse. Pots were rotated and exchanged every three days to avoid the influences of positions. Seedlings were watered and weighed every day to maintain soil moisture at 70–80% of field capacity.

### Data collection and analyses

The initial size (basal diameter of stem) was measured for each plant individual before any treatments. After early and late treatments, three individuals per species per treatment were randomly chosen on a sunny morning (from 09:30 to 11:00), to measure photosynthetic physiological traits on one fully expanded adult leaf, with Li-6400 photosynthesis instrument (with air temperature of 20 ± 5 °C, CO_2_ concentration of 380 ± 5 µmol·m^− 1^, relative air humidity of 60 ± 5%, and photoactive radiation of 600 µmol·m^− 2^·s^− 1^): net photosynthetic rate (Pn), transpiration rate (Tr), stomatal conductance (Cond), and intercellular CO_2_ concentration (Ci). Then these plants were used to measure biochemical traits on one fully expanded adult leaf, including soluble protein content, soluble sugar content [29], and free proline content [30]; another three individuals per species per treatment were randomly chosen to determine malondialdehyde content [31], catalase activity [32], peroxidase activity [33], and superoxide dismutase activity [32] on one fully expanded adult leaf for each individual.

For morphological and biomass traits, another three individuals per species per treatment were used to measure the following traits after early and late treatments: the length and basal diameter of stem, and leaf area of the largest leaf. Each plant individual was then separated into roots and shoots, dried at 105 °C for 1 h and then dried at 70 °C to constant weight and weighed. Total biomass was the total of shoot biomass and root biomass. Late growth (LG) for all morphological and biomass traits was calculated with the formula [14] as:

1 $$\mathrm{LG}=\left(Y-X\right)/\;X$$

where *X* is the mean trait value at the end of an early treatment, and *Y* is the mean trait value in a late treatment after the same early treatment. For example, for relative growth in total mass (LG_TM_) of a species in late moderate shading after early constant full-light experience, *X* is its mean total biomass at the end of early full light treatment, and *Y* is its mean total biomass in late moderate shading after early full light experience. The index of composite late growth (LG_C_) was calculated by averaging the LG of all five traits including stem basal diameter, stem length, maximum leaf area, shoot biomass, and root biomass.

For each of all kinds of traits (photosynthetic physiological, biochemical, morphological and biomass traits), we also calculated Diff-values (Diff-Y, difference in late performance of a trait between temporally heterogeneous and homogeneous light experiences) for each species and each late treatment, to compare effects of early temporally heterogeneous light experience between different species or late conditions [14], with the formula as:

2 $$\mathrm{Diff}-Y=Y_{het\;}-Y_{hom}$$

where *Y*_*het*_ is the mean trait value in a late condition after fluctuating light experience, and *Y*_*hom*_ is the mean trait value under the same late condition after constant full light or moderate shading experience. Since there were two constant light treatments in control, there were two Diff-values per trait per species in each late treatment.

For all morphological and biomass traits, three-way ANCOVAs were conducted for effects of early treatment, late treatment, species, and their interactions, with the initial size (stem basal diameter) nested in species as a covariate. For late growth of morphological and biomass traits, photosynthetic physiological and biochemical traits, three-way ANOVAs were conducted for effects of early treatment, late treatment, species, and their interactions. Diff-values of all traits were analyzed by two-way ANOVAs for effects of late treatment, species and their interactions. One-way ANCOVA or ANOVA was then used to analyze effects of early treatment, late treatment, or species on all traits within each and across all of the other treatments. Multiple comparisons used the Least Significant Difference (LSD) method (*P* < 0.05) in General Linear Program (GLM).

## Results

### Morphological performance and late growth

**Fig. 2:**
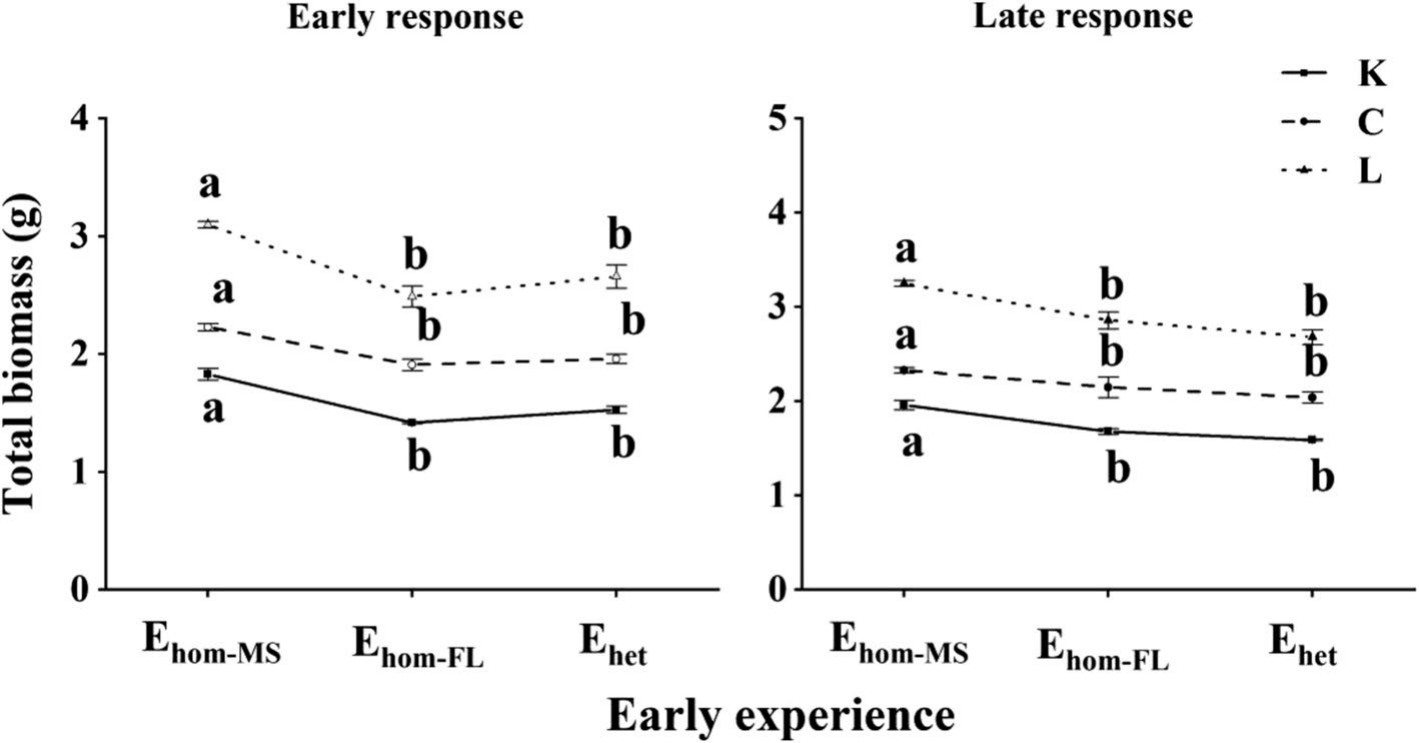
Mean total biomass (± SE) of *Kmeria septentrionalis* (K), *Celtis sinensis* (C), *Lithocarpus glaber* (L) after the 1st round of homogeneous moderate shading (E_hom−MS_), full light (E_hom−FL_), and temporally heterogeneous light (E_het_) treatments (early experience) and for all late conditions after the 2nd round (late response) for plants with different early experiences. Different lowercase letters indicate differences between early treatments (the 1st round; *P* < 0.05)

Effects of species, early treatment and late treatment were significant for most morphological traits and their late growth, interactions between species and early treatment, between species and late treatment and between early treatment and late treatment were significant for late growth in total biomass (LG_TM_) and mean late growth of all the other traits (or composite late growth, LG_C_; Table 1 and Tables S1, S2). After early treatments (the 1st round), compared to moderate shading conditions (E_hom−MS_), both temporally heterogeneous light (E_het_) and full light (E_hom−FL_) conditions reduced mean shoot mass, root mass, and total mass for all species (LSD, *P* < 0.05), with no differences between effects of E_hom−FL_ and E_het_ (Fig. 2 and Fig. S1). After late treatments (the 2nd round), compared to E_hom−MS_, E_het_ and E_hom−FL_ reduced mean shoot mass, root mass, and total mass of all species across all or in each of late light conditions (*P* < 0.05), with no differences between effects of E_hom−FL_ and E_het_ (Fig. 2 and Fig. S1).

**Table 1:** *F*-values from three-way ANCOVA for log-transformed total mass (TM) and ANOVA for late growth of total mass (LG_TM_), composite late growth (LG_C_, mean late growth for traits of stem basal diameter, stem length, maximum leaf area, shoot biomass and root biomass), malondialdehyde content (MDA), catalase (CAT), superoxide dismutase (SOD) and peroxidase (POD) activity, showing effects of species (SP), early treatment (ET), and late treatment (LT) and their interactions, with log10 (initial size [IS]) nested in the species effect as a covariate in ANCOVA

Source of variance	Df	Log_10_ TM	LG_TM_	LG_C_	MDA	CAT	SOD	POD
Log_10_ (IS)	1	0.82						
SP	2	1125.48^***^	354.57^***^	128.27^***^	275.18^***^	216.82^***^	47.57^***^	152.71^***^
ET	2	101.54^***^	294.27^***^	74.39^***^	143.86^***^	206.23^***^	116.20^***^	149.23^***^
LT	2	4.10^*^	390.62^***^	122.74^***^	1247.56^***^	318.57^***^	11.20^***^	272.47^***^
SP × ET	4	5.36^**^	20.85^***^	7.30^***^	5.35^*^	26.64^***^	20.73^***^	54.03^***^
SP × LT	4	0.22	22.11^***^	7.81^***^	13.78^***^	21.68^***^	1.65	6.70^**^
ET × LT	4	0.87	58.83^***^	17.64^***^	33.38^***^	64.05^***^	3.44^*^	46.55^***^
SP × ET × LT	8	0.06	6.97^***^	1.42	2.36^*^	5.43^**^	3.13^*^	17.76^***^

Significance levels: * *P* < 0.05, ** *P* < 0.01, *** *P* < 0.001

**Fig. 3:**
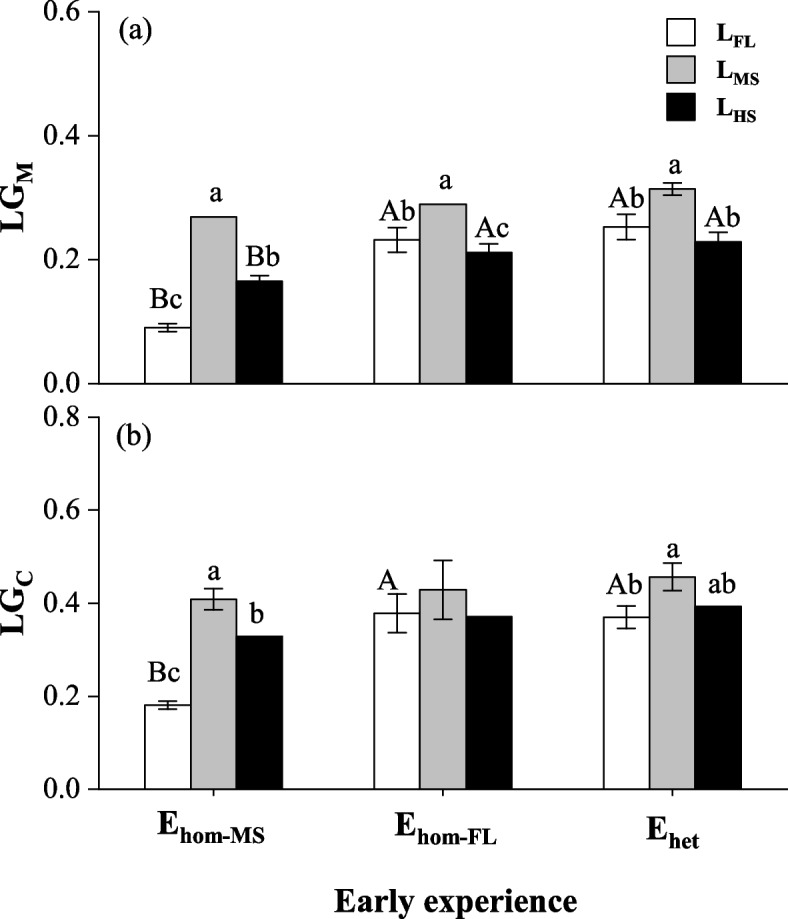
Mean values (± SE) of late growth in total biomass (LG_M_) and composite late growth (LG_C_, mean late growth for traits of stem basal diameter, stem length, maximum leaf area, shoot biomass and root biomass) in late full light (L_FL_), moderate shading (L_MS_), and heavy shade (L_HS_) conditions for *Kmeria septentrionalis* (K), *Celtis sinensis* (C), *Lithocarpus glaber* (L) with early experiences of homogeneous moderate shading (E_hom−MS_), full light (E_hom−FL_), and temporally heterogeneous light (E_het_) conditions. Different lowercase letters indicate significant differences between late conditions within early experiences, and different uppercase letters indicate significant differences between early experiences within late conditions (*P* < 0.05)

Compared to E_hom−MS_, E_het_ increased the relative growth (LG_TM_ and LGc) by an average of 31.73% (LSD, *P* < 0.001), and E_hom−FL_ increased them by average 20.44%, across all species and all late conditions (*P* < 0.05), with greater improvement by E_het_ versus E_hom−FL_ (*P* < 0.05; Fig. 3a, b); E_het_ and E_hom−FL_ also enhanced LG of shoot mass, root mass and total mass, and LG_C_ for each species in each late light conditions (*P* < 0.05; Fig. S2 and Fig. S3).

### Photosynthetic physiological and biochemical performances

**Fig. 4:**
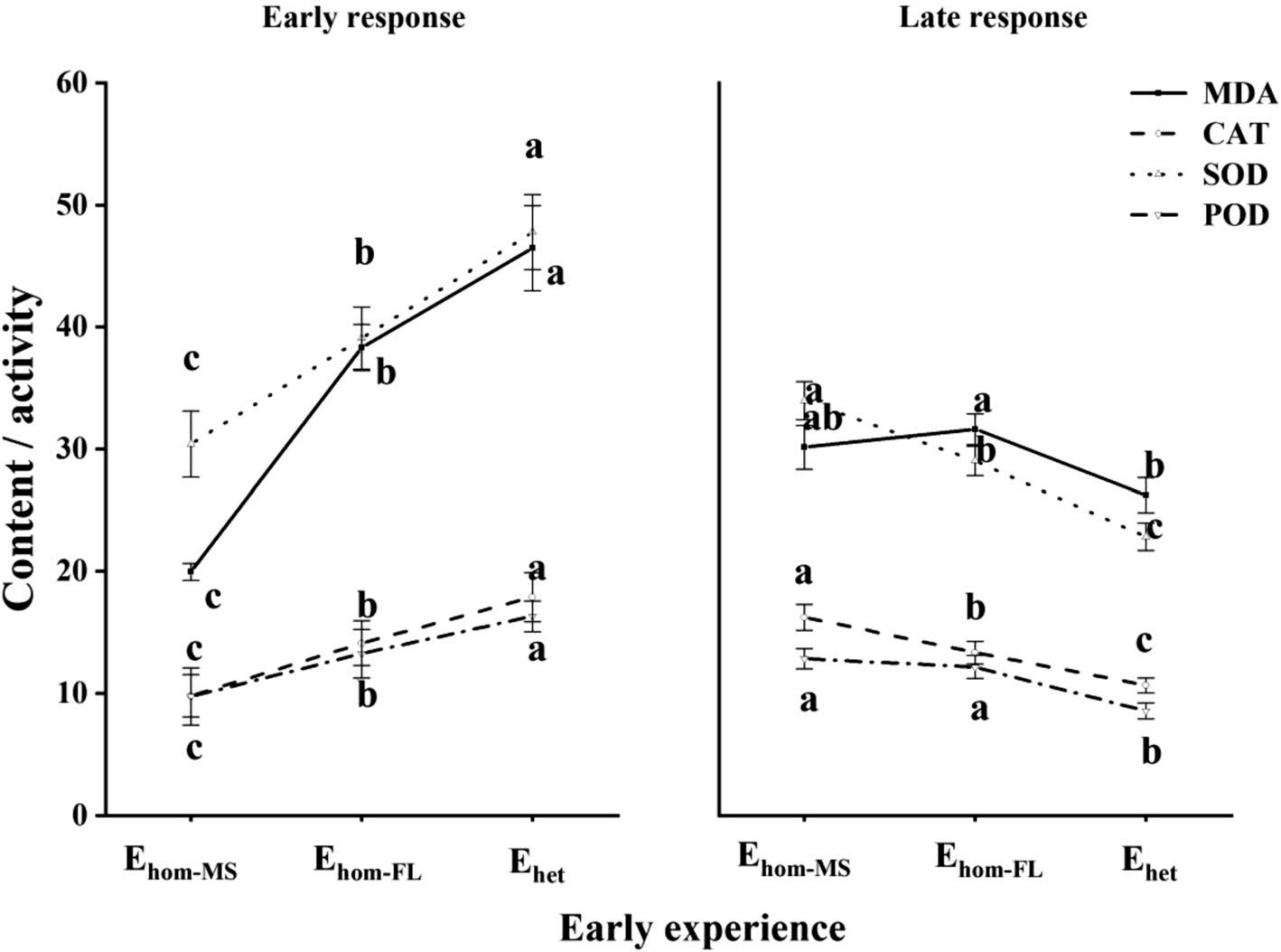
Mean values (± SE) of malondialdehyde content (MDA), catalase (CAT), superoxide dismutase (SOD) and peroxidase (POD) activity of *Kmeria septentrionalis* (K), *Celtis sinensis* (C), *Lithocarpus glaber* (L) after the 1st round of homogeneous moderate shading (E_hom−MS_), full light (E_hom−FL_), and temporally heterogeneous light (E_het_) treatments (early experience) and for all late conditions after the 2nd round (late response) for plants with different early experiences. Different lowercase letters indicate differences between early treatments (the 1st round; *P* < 0.05)

Effects of early treatment, late treatment, and species were significant for all traits, interaction between early treatment and late treatment was significant for most traits, and interaction between early treatment and species was also significant for most biochemical traits (Table 1 and Table S3). Across or for all species and late conditions, the levels of malondialdehyde content (MDA), and catalase (CAT), superoxide dismutase (SOD) and peroxidase (POD) activity of plants with different early experiences tended to range as: E_hom−MS_ < E_hom−FL_ < E_het_, after early treatments (LSD, *P* < 0.05), whereas tended to range as: E_hom−MS_ > E_hom−FL_ > E_het_, after late treatments (Fig. 4 and Figs. S4, S5). After early treatments, for all species, compared to E_hom−MS_, E_het_ and E_hom−FL_ reduced photosynthetic rate (Pn), transpiration rate (Tr) and stomatal conductance (Cond) for all species, but enhanced intercellular CO_2_ concentration (Ci, LSD, *P* < 0.05), with no differences between E_hom−FL_ and E_het_ (Fig. S6). After late treatments, compared to E_hom−MS_, E_hom−FL_ and E_het_ was more likely to enhance Pn, Tr and Cond of plants (*P* < 0.05), but E_het_ was more likely to reduced Ci of plants (*P* < 0.05), especially in late full light and heavy shading conditions (Fig. S6).

### Diff-value in traits

**Fig. 5:**
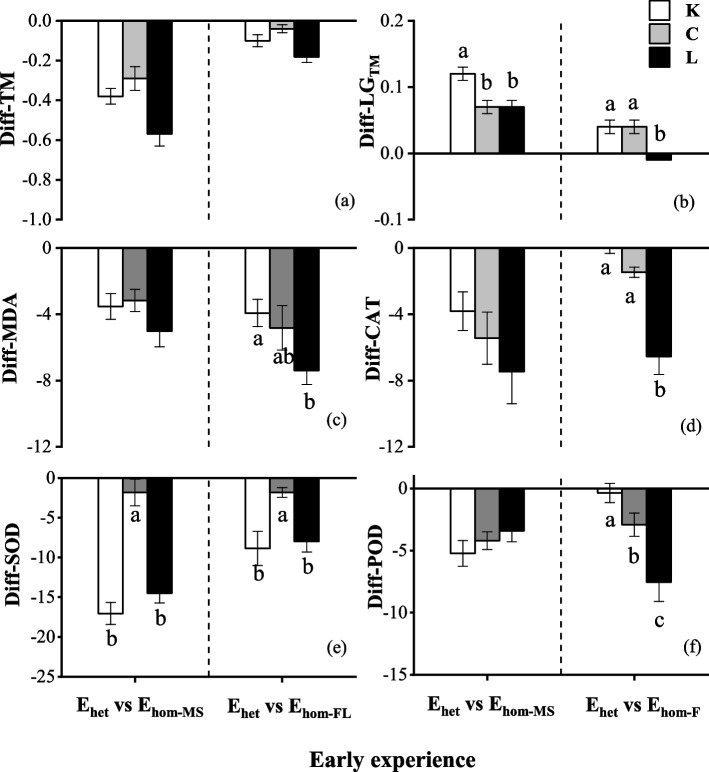
Mean difference values (± SE) due to the effect of early heterogeneous light relative to homogeneous moderate shading (E_hom−MS_) or full light (E_hom−FL_) experience in total biomass (Diff-TM), late growth of total biomass (Diff-LG_TM_), malondialdehyde content (Diff-MDA), catalase (Diff-CAT), superoxide dismutase (Diff-SOD) and peroxidase (Diff-POD) activity for *Kmeria septentrionalis* (K), *Celtis sinensis* (C), *Lithocarpus glaber* (L) across all late treatments. Different lowercase letters indicate differences between species (*P* < 0.05)

Effects of species and late conditions were significant on Diff-values (difference in mean trait values due to early experience) for most traits, and effects of interaction between species and late treatments was also significant for relative growth of total mass (Diff-LG_TM_) and superoxide dismutase activity (Diff-SOD; Table 2). Across all late conditions, Diff-TM, Diff- MDA, Diff- CAT, Diff- SOD, and Diff- POD were either negative or insignificant, but Diff-LG_TM_ were mostly positive (Fig. 5). Comparing diff-values among species across all late conditions, *K. septentrionalis* had higher positive Diff-LG_TM_ due to E_het_ vs. E_hom−MS_ than other species (*P* < 0.05), and *L. glaber* had lower Diff-LG_TM_ due to E_het_ vs. E_hom−FL_ than other species (*P* < 0.05; Fig. 5b), and lower Diff-CAT, Diff-MDA and Diff-POD due to E_het_ vs. E_hom−FL_ than the other two species (*P* < 0.05; Fig. 5c, d, f), and *C. sinensis* had higher Diff-SOD due to E_het_ vs. E_hom−MS_ or E_het_ vs. E_hom−FL_ than the other two species (*P* < 0.05; Fig. 5e).

**Table 2:** * F*-values from two-way ANOVA for effects of late treatment (LT), and species (SP) and their interactions on the Diff-values (difference in mean trait values due to effects of early temporally heterogeneous light (E_het_) vs. control (E_hom−MS_ or E_hom−FL_) experience for total mass (Diff-TM), late growth of total mass (Diff-LG_TM_), malondialdehyde content (Diff-MDA), catalase (Diff-CAT), superoxide dismutase (Diff-SOD) and peroxidase (Diff-POD) activity

Type	Source	Df	TM	LG_TM_	MDA	CAT	SOD	POD
E_het_ vs. E_hom−MS_	SP	2	21.07^***^	77.01^***^	4.36^*^	45.85^***^	14.18^***^	3.62^*^
	LT	2	2.08	9.15^**^	21.45^***^	7.32^**^	105.90^***^	31.34^***^
	SP × LT	4	0.02	34.62^***^	2.47^*^	0.66	7.27^**^	0.36
E_het_ vs. E_hom−FL_	SP	2	23.30^***^	29.33^***^	6.43^**^	12.65^***^	89.62^***^	98.32^***^
	LT	2	0.09	3.48^*^	13.87^***^	3.86^*^	7.15^**^	144.07^***^
	SP × LT	4	0.76	11.04^***^	1.29	5.17^**^	12.93^***^	42.38^***^

Significance levels: * *P* < 0.05, ** *P* < 0.01, *** *P* < 0.001

## Discussion

### Effects of early experience with temporally heterogeneous light availability

Temporally heterogeneous environments can lead to an increase of plasticity [3-6], which has rarely been supported by direct evidence. This study provided such evidence that temporally heterogeneous light experience induced variations in a great number of different traits [10, 34]. Compared to constant full light experience, early exposure to temporally heterogeneous light conditions increased the activity of a series of enzymes and did not affect most of other traits immediately, and improved late growth in morphological and biomass traits with decrease or no changes in most of the other traits finally. It suggested temporally heterogeneous light experience can improve plant performance via different mechanisms at different stages. Early exposure to stressful conditions can improve later tolerance for stress [35-37], which is also called priming effects [38]. It is reported that alternate inundation and drought experience has been harmful for plants immediately, but can improve their late growth in biomass later [13]. Our results demonstrated that early experience with such fluctuating conditions may not be simply harmful immediately, but it can induce active biochemical responses (increase in performance), to support later morphological and biomass responses. Therefore, temporal heterogeneous experience can also be beneficial immediately in terms of biochemical performance. Plants may not only have the intelligence to adjust their subsequent performance according to previous environmental experiences, but also are able to make decisions and adjustments very rapidly at the time of exposure to an environmental condition.

Moreover, it is also noteworthy that different kinds of traits did not respond to temporally heterogeneous light treatment synchronously, and biochemical adjustments occurred ahead of morphological and biomass responses. Early heterogeneous experience can induce compensatory growth in plants [39-41], which may have been achieved via early responses at biochemical level. Plants experiencing fluctuating light conditions should have received unstable or unreliable environmental cues, which made them less able to predict the future situations accordingly. Meanwhile, plastic responses of morphological and biomass traits are more irreversible and associated with higher production and maintaining costs than other kinds of traits [42, 43]. If they produce plastic responses in these traits when environmental signals are unreliable, plants may face higher risks of improper responses due to failing predictions, leading to the loss of both early costs and no later profits. However, biochemical responses may cause lower costs, or be connected to more flexible strategies of reaction, thus more profitable in this situation. Improved biochemical performance may be helpful for producing morphological and biomass responses subsequently, irrespective of the light conditions plants will expose to. Therefore biochemical plasticity may bring about higher profits than costs, and plants are more likely to rely on plastic responses of biochemical traits than morphological responses in dealing with short-term or unpredictable stressful events [44, 45]. Consequently, plastic responses at biochemical level can be more beneficial for plants in highly spatial or temporal heterogeneous environments than morphological responses.

### Effects of early moderate shading experience

Our results showed that compared to those with constant full light experience, plants experiencing with constant moderate shading conditions showed higher performance in photosynthetic physiological and biomass traits and lower biochemical performance, immediately after experience; but they had decreased performance in photosynthetic physiological traits and late growth in biomass in the end. It suggests effects of homogeneous shading experience contrast with that of heterogeneous light experience: it induced active responses of morphological and photosynthetic physiological traits in the first place, which resulted in costs of lower growth potential later although, leading to stable performance in final. This should be because in reaction to the relatively stable or reliable signals from constant shading experience, plants will be more likely to produce relatively irreversible morphological responses, in spite of its higher costs. It appears that when future environmental conditions are relatively predictable according to current signals, they will prefer to make adjustments in morphological and biomass aspects as early as possible, which is at the cost of future growth potential though. Since morphological and biomass performance are more crucial for plant size than other traits, plants with enhanced performance in these traits should more likely grow into large individuals and survive than otherwise [46]. Morphological and biomass responses thus should be more direct and cost-saving than other kinds of responses. However, in face of unstable or unpredictable stress or environmental challenges, plants may take greater risks of high costs and low profits if they produce responses of morphological and biomass traits [43], which may not match with the future environments [18]. Not matching signifies that plants have failed in making correct or accurate prediction, decision and reaction initially, the loss of which will be unable to afford to them.

Meanwhile, active responses of photosynthetic physiological traits should have supported morphological and biomass responses. Sometimes, physiological plasticity may even contribute more to the ability of invasion of species than morphological plasticity [47]. This might be because that the costs of physiological plasticity are lower than that of morphological plasticity, although this may simply be because physiological changes are often invisible, while morphological plasticity involves production of new parts [47, 48]. Nevertheless, the production of both photosynthetic physiological and morphological responses at early stage must have caused substantial costs, which should have directly led to reduced late growth potential of biomass. This phenomenon has been considered as a “life-history limit” to plasticity [13, 43, 49]. On the other hand, decreased late growth in biomass also reflected the costs due to production of plastic responses at early stage [13]. Costs of plasticity are often difficult to detect [50]. It may be because of the lack of the perspective of meta-plasticity or variable plasticity over different stages of plant growth. By virtue of such perspective, we may be able to better detect and understand the costs and benefits of plasticity, as well as the intelligence of plants [13].

In spite of significant costs, plants with early constant shading experience can also succeed through rapid early morphological and biomass responses, compared with those with constant full-light experience. It suggests plants are able to use diverse strategies to deal with various environmental changes, all of which can lead to the final success in survival and persistence.

### Species from different ranges of habitats

Studies have found that species from karst habitats can better adapt to higher heterogeneous environments than other species, due to greater plasticity in traits [51, 52]. However, few studies have provided direct evidence by comparing karst species with species from other habitat ranges. In this study, we showed species from different habitat ranges can all benefit from effects of temporally heterogeneous light experience, and beneficial effects differ in extent for different species: it was more beneficial for karst species of *Kmeria septentrionalis* than for the other two species, the least beneficial for non-karst species of *Lithocarpus glaber*, especially in late heavy shading conditions. The ability of species to utilize early experience can be associated with their habitat ranges. The environments of karst ecosystems are highly heterogeneous both spatially and temporally, indicating variations in various abiotic factors, such as soil moisture, nutrients, and light conditions [24, 53]. Plant species with a long-term history of acclimation to karst habitats may have developed stronger capacity to deal with such environmental heterogeneity, thus can better adapt to or benefit from fluctuating light experience. In contrast, species from non-karst habitats may have lower chance to encounter highly heterogeneous environments, thus lack the ability to cope with fluctuating experience.

## Conclusions

Our results demonstrated that plants are able to adopt appropriate strategies to deal with either fluctuating light or constant shading experience, and can benefit from such experience in different ways, both leading to their success in final performance. Effects of temporally heterogeneous vs. homogeneous light conditions are contrasting: the former induced higher level of biochemical traits immediately and improved late growth in biomass traits, while the latter decreased biochemical traits, improved physiological and morphological traits immediately with lower late growth potential. Morphological and biomass adjustments should be the most direct and cost-saving than the other kinds of responses for improving plant size and survival, but also are less reversible and associated with higher costs than other kinds of traits. When future environments are more predictable, plants will prefer to make these adjustments as early as possible, although at the cost of decreased future growth potential. In face of unpredictable environments, plants may avoid taking greater risks of high costs and no profits by producing these responses and final failure, and prefer to produce biochemical responses as early as possible, which should be beneficial in face of whatever future environments.

Typical karst species may be more able to benefit from temporally heterogeneous experience, compared to generalists and non-karst species. Since only three species from different ranges of habitats have been used for comparison, conclusions might be limited in applying to a wide range of karst or non-karst species. Analogous studies using a greater amount of karst species in comparison with non-karst species may provide further evidence for our hypothesis. Research on effects of temporally heterogeneous experience on plant later responses should have shed light on the capacity of plants to modulate performance and response in their lifetime, or variable plasticity or meta-plasticity. Such ability of adjusting responses should be of greater significance than short-term plastic responses for plant adaptation. In the perspective of meta-plasticity over different stages of plant growth, we can better detect and understand the costs and benefits of plasticity, as well as the intelligence of plants.

## Supplementary Information

**Additional file 1: Fig. S1. **Mean values (±SE) of stem basal diameter (SD), stem length (SL), maximum leaf area (LA_m_), shoot biomass (SM) and root biomass (RM) of *Kmeria septentrionalis* (K),* Celtis sinensis* (C), *Lithocarpus glaber* (L) in homogeneous moderate shading (E_hom-MS_), full light (E_hom-FL_) and temporally heterogeneous light conditions (E_het_) (early experience), and in late full light (L_FL_), moderate shading (L_MS_), heavy shading (L_HS_) after different early experiences (late response). Different lowercase letters indicate differences between early treatments for each species in early response and between late treatments within the same early experiences in late response, different uppercase letters indicate differences between early experiences for each species within the same late treatments in late response (*P* < 0.05). **Fig. S2.** Mean values (±SE) of late growth (LG) of stem basal diameter (LG_SD_), stem length (LG_SL_), maximum leaf area (LG_LAm_), shoot biomass (LG_SM_) and root biomass (LG_RM_) in late full light (L_FL_), moderate shading (L_MS_), heavy shading (L_HS_) treatments for *Kmeria septentrionalis* (K),* Celtis sinensis* (C), *Lithocarpus glaber* (L) with early experiences of homogeneous moderate shading (E_hom-MS_), full light (E_hom-FL_) and temporally heterogeneous light (E_het_) conditions. Different lowercase letters indicate differences between late treatments for each species within the same early experiences, different uppercase letters indicate differences between early experiences for each species within the same late treatments (*P* < 0.05). **Fig. S3.** Composite late growth (LG_C_) or mean late growth for stem basal diameter (SD), stem length (SL), maximum leaf area (LA_m_), shoot biomass (SM) and root biomass (RM) in late full light (L_FL_), moderate shading (L_MS_) and heavy shading (L_HS_) for *Kmeria septentrionalis* (K),* Celtis sinensis* (C), *Lithocarpus glaber* (L) with early experiences of homogeneous moderate shading (E_hom-MS_), full light (E_hom-FL_) and temporally heterogeneous light (E_het_) conditions. Different letters indicate differences between early experiences for each species within the same late treatments (*P* < 0.05). **Fig. S4.** Mean contents (±SE) of soluble protein (SP), soluble sugar (SS), free proline (Pro) for *Kmeria septentrionalis* (K),* Celtis sinensis* (C), *Lithocarpus glaber* (L) in homogeneous moderate shading (E_hom-MS_), full light (E_hom-FL_) and temporally heterogeneous light conditions (E_het_) (early experience, in the first round of treatments), and in late full light (L_FL_), moderate shading (L_MS_), heavy shading (L_HS_) after different early experiences (late response, in the second round of treatments). Different lowercase letters indicate differences between early treatments for each species in early response and between late treatments within the same early experiences in late response, different uppercase letters indicate differences between early experiences for each species within the same late treatments in late response (*P* < 0.05). **Fig. S5.** Mean values (±SE) of malondialdehyde content (MDA), catalase (CAT), superoxide dismutase (SOD) and peroxidase (POD) activity of *Kmeria septentrionalis* (K),* Celtis sinensis* (C), *Lithocarpus glaber* (L) in homogeneous moderate shading (E_hom-MS_), full light (E_hom-FL_) and temporally heterogeneous light conditions (E_het_) (early experience), and in late full light (L_FL_), moderate shading (L_MS_), heavy shading (L_HS_) after different early experiences (late response). Different lowercase letters indicate differences between early treatments for each species in early response and between late treatments within the same early experiences in late response, different uppercase letters indicate differences between early experiences for each species within the same late treatments in late response (*P* < 0.05). **Fig. S6.** Mean values (±SE) of net photosynthesis rate (Pn), transpiration rate (Tr), stomatal conductance (Cond), and intercellular CO_2_ concentration (Ci) of *Kmeria septentrionalis* (K),* Celtis sinensis* (C), *Lithocarpus glaber* (L) in homogeneous moderate shading (E_hom-MS_), full light (E_hom-FL_) and temporally heterogeneous light conditions (E_het_) (early experience), and in late full light (L_FL_), moderate shading (L_MS_), heavy shading (L_HS_) after different early experiences (late response). Different lowercase letters indicate differences between early treatments for each species in early response and between late treatments within the same early experiences in late response, different uppercase letters indicate differences between early experiences for each species within the same late treatments in late response (*P* < 0.05). **Table S1. ***F*-values from three-way ANCOVA for effects of species (SP), early treatment (ET), and late treatment (LT) and their interactions on log-transformed stem basal diameter (SD), stem length (SL), maximum leaf area (LA_m_), shoot biomass (SM) and root mass (RM), for plants with two rounds of treatments, with log_10_(Initial size [IS]) nested in the species as a covariate. Significance levels:* *P* < 0.05, ** *P* < 0.01, *** *P* < 0.001. **Table S2.** *F*-values from three-way ANOVA for the effects of species (SP), early treatment (ET), and late treatment (LT) and their interactions on late growth in stem basal diameter (LG_SD_), stem length (LG_SL_), maximum leaf area (LG_LAm_), shoot biomass (LG_SM_) and root biomass (LG_RM_), for plants with two rounds of treatments. **Table S3.** *F*-values from three-way ANOVA for the effects of species (SP), early treatment (ET), and late treatment (LT) and their interactions on photosynthesis rate (Pn), transpiration rate (Tr), stomatal conductance (Cond), intercellular CO_2_ concentration (Ci), soluble protein (SP), soluble sugar (SS), and free proline (Pro) content, for plants with two rounds of treatments

## Data Availability

The datasets supporting the conclusions of this article are included within the article and its additional files.

## Abbreviations

ET: Early treatment
E_het_: Early temporally heterogeneous light
E_hom-MS_: Early temporally homogeneous moderate shading
E_hom-FL_: Early temporally homogeneous full light
LT: Late treatment
L_FL_: Late full light
L_MS_: Late moderate shading
L_HS_: Late heavy shading
TM: Total mass
MDA: Malondialdehyde content
CAT: Catalase activity
SOD: Superoxide dismutase activity
POD: Peroxidase activity
Pn: Photosynthetic rate
Tr: Transpiration rate
Cond: Stomatal conductance
Ci: Intercellular CO_2_ concentration
LG: Late growth
LG_TM_: Late growth in total biomass
LG_C_: Composite late growth
Diff-TM: Difference in total mass
Diff-LG_TM_: Difference in late growth of total mass
Diff-MDA: Difference in late performance of malondialdehyde content
Diff-CAT: Difference in late performance of catalase activity
Diff-SOD: Difference in late performance of superoxide dismutase activity
Diff-POD: Difference in late performance of peroxidase activity
SP: Species
K: *Kmeria septentrionalis*
C: *Celtis sinensis*
L: *Lithocarpus glaber*
