# Supplementary material for: Contrasting effects of experiencing temporally heterogeneous light availability versus homogenous shading on plant subsequent responses to light conditions
Source: BMC Plant Biol. 2023 May 3;23:232. doi: 10.1186/s12870-023-04229-4 (PMC10155447; doi:10.1186/s12870-023-04229-4)
Supplement: Supplementary file 1 — Additional file 1: Fig. S1. Mean values (±SE) of stem basal diameter (SD), stem length (SL), maximum leaf area (LAm), shoot biomass (SM) and root biomass (RM) of Kmeria septentrionalis (K), Celtis sinensis (C), Lithocarpus glaber (L) in homogeneous moderate shading (Ehom-MS), full light (Ehom-FL) and temporally heterogeneous light conditions (Ehet) (early experience), and in late full light (LFL), moderate shading (LMS), heavy shading (LHS) after different early experiences (late response). Different lowercase letters indicate differences between early treatments for each species in early response and between late treatments within the same early experiences in late response, different uppercase letters indicate differences between early experiences for each species within the same late treatments in late response (P < 0.05). Fig. S2. Mean values (±SE) of late growth (LG) of stem basal diameter (LGSD), stem length (LGSL), maximum leaf area (LGLAm), shoot biomass (LGSM) and root biomass (LGRM) in late full light (LFL), moderate shading (LMS), heavy shading (LHS) treatments for Kmeria septentrionalis (K), Celtis sinensis (C), Lithocarpus glaber (L) with early experiences of homogeneous moderate shading (Ehom-MS), full light (Ehom-FL) and temporally heterogeneous light (Ehet) conditions. Different lowercase letters indicate differences between late treatments for each species within the same early experiences, different uppercase letters indicate differences between early experiences for each species within the same late treatments (P < 0.05). Fig. S3. Composite late growth (LGC) or mean late growth for stem basal diameter (SD), stem length (SL), maximum leaf area (LAm), shoot biomass (SM) and root biomass (RM) in late full light (LFL), moderate shading (LMS) and heavy shading (LHS) for Kmeria septentrionalis (K), Celtis sinensis (C), Lithocarpus glaber (L) with early experiences of homogeneous moderate shading (Ehom-MS), full light (Ehom-FL) and temporally heterogeneo [file 12870_2023_4229_MOESM1_ESM.docx]

**Supporting information**

Additional supporting information may be found in the online version of this article. Fig. S1-S6, Tables S1-S3.

**
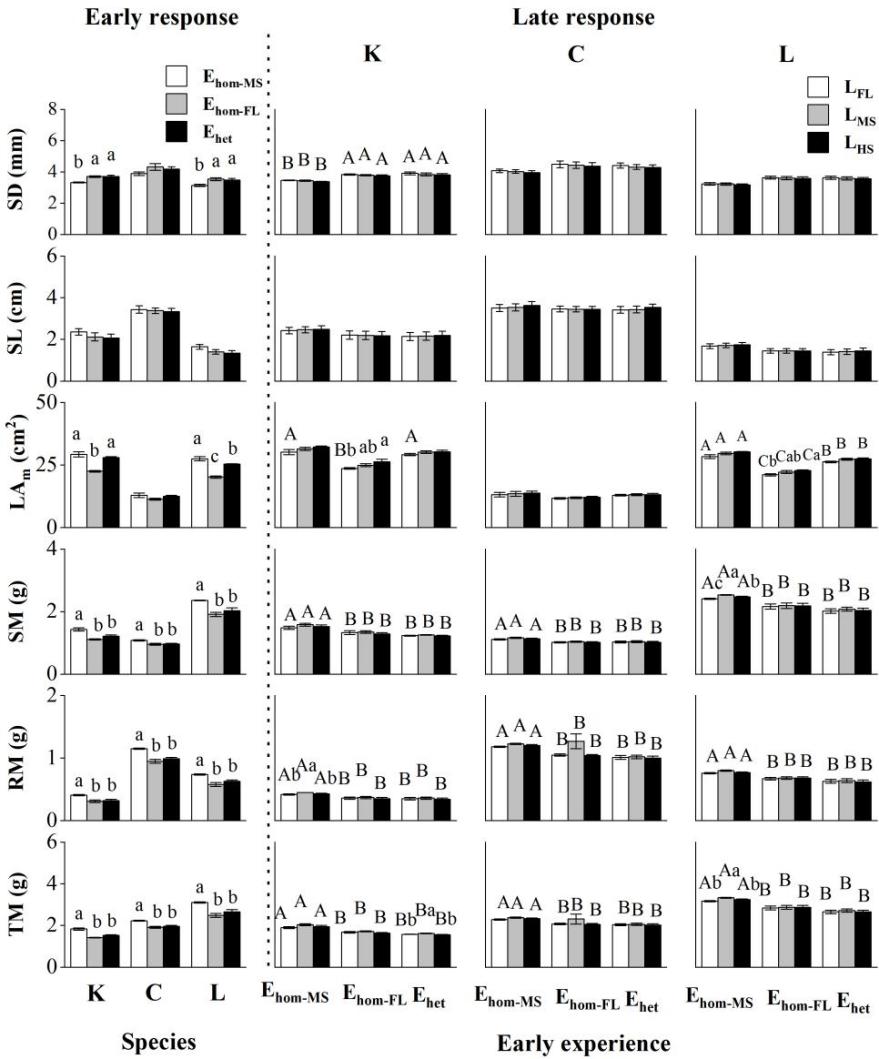
Fig. S1** Mean values (±SE) of stem basal diameter (SD), stem length (SL), maximum leaf area (LA_m_), shoot biomass (SM) and root biomass (RM) of *Kmeria septentrionalis* (K), *Celtis sinensis* (C), *Lithocarpus glaber* (L) in homogeneous moderate shading (E_hom-MS_), full light (E_hom-FL_) and temporally heterogeneous light conditions (E_het_) (early experience), and in late full light (L_FL_), moderate shading (L_MS_), heavy shading (L_HS_) after different early experiences (late response). Different lowercase letters indicate differences between early treatments for each species in early response and between late treatments within the same early experiences in late response, different uppercase letters indicate differences between early experiences for each species within the same late treatments in late response (*P* < 0.05).

**
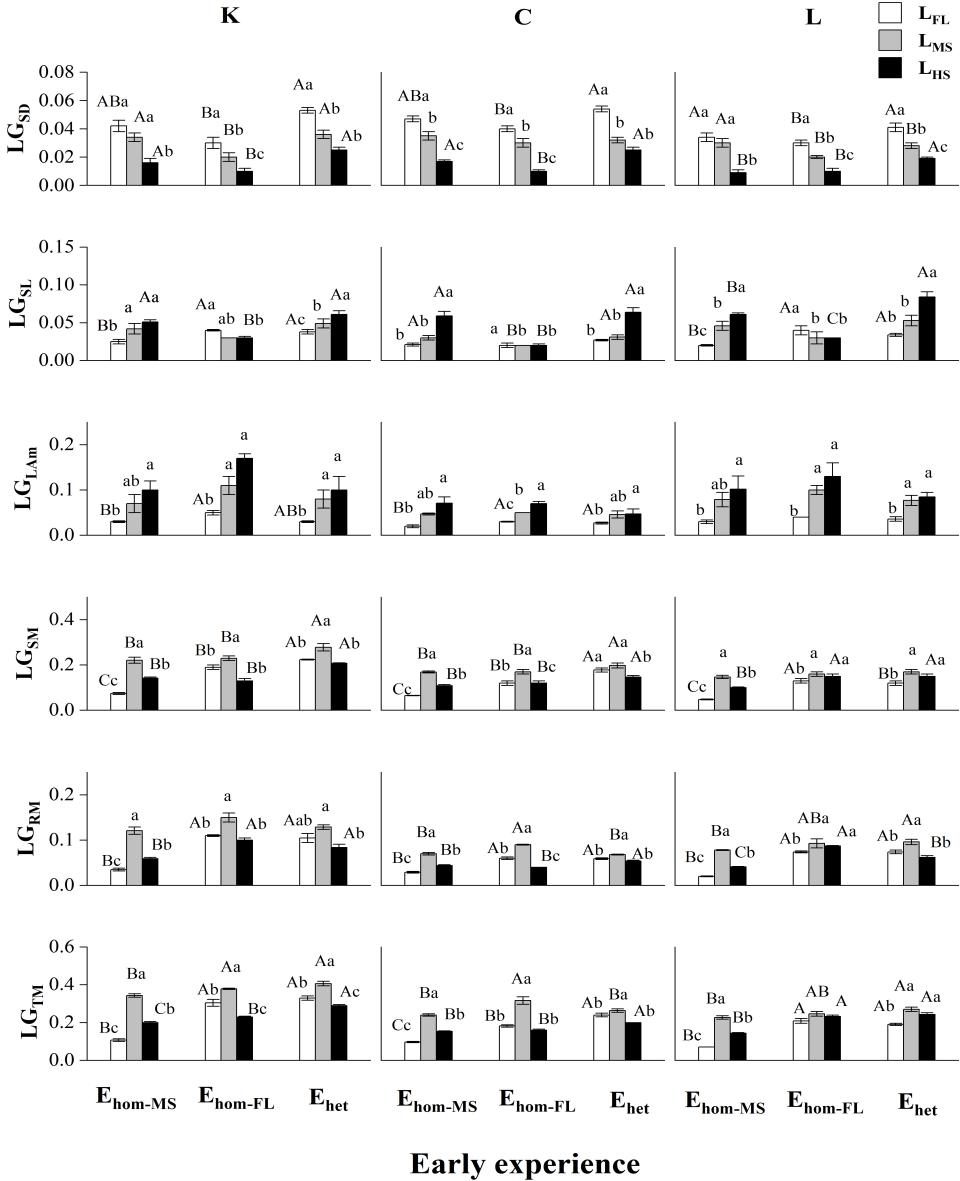
Fig. S2** Mean values (±SE) of late growth (LG) of stem basal diameter (LG_SD_), stem length (LG_SL_), maximum leaf area (LG_LAm_), shoot biomass (LG_SM_) and root biomass (LG_RM_) in late full light (L_FL_), moderate shading (L_MS_), heavy shading (L_HS_) treatments for *Kmeria septentrionalis* (K), *Celtis sinensis* (C), *Lithocarpus glaber* (L) with early experiences of homogeneous moderate shading (E_hom-MS_), full light (E_hom-FL_) and temporally heterogeneous light (E_het_) conditions. Different lowercase letters indicate differences between late treatments for each species within the same early experiences, different uppercase letters indicate differences between early experiences for each species within the same late treatments (*P* < 0.05).

**
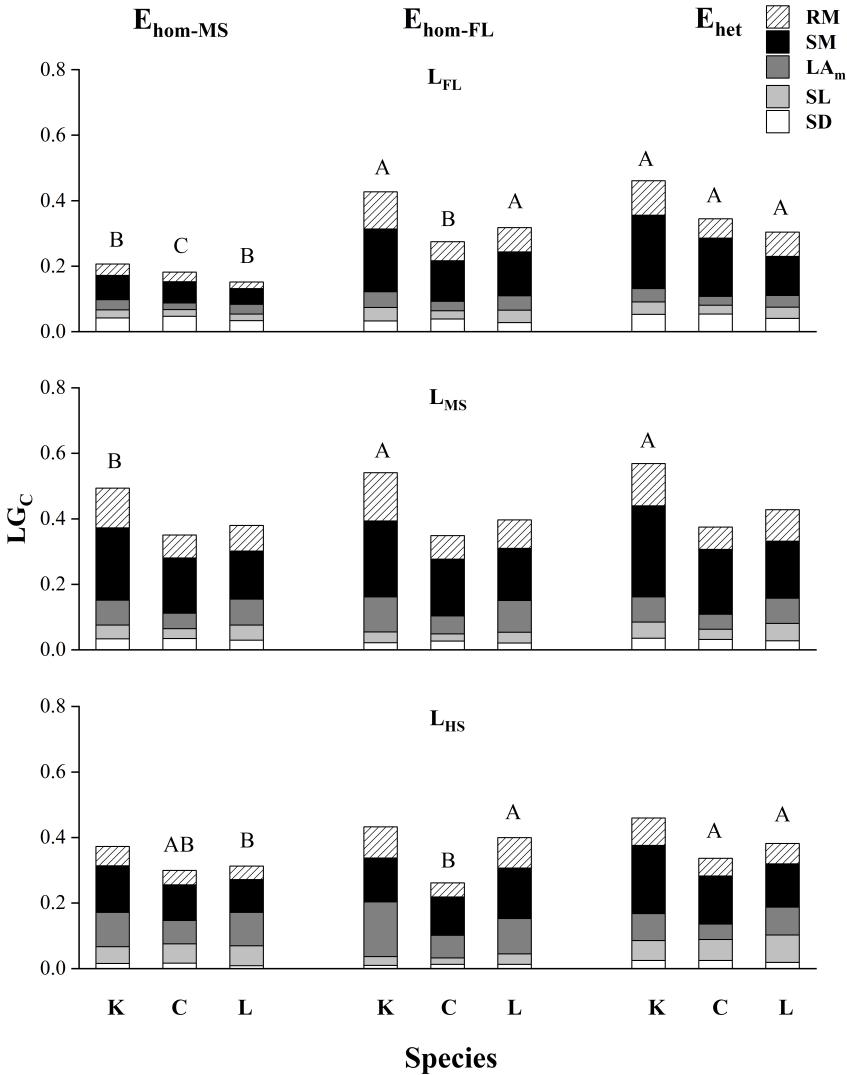
Fig. S3** Composite late growth (LG_C_) or mean late growth for stem basal diameter (SD), stem length (SL), maximum leaf area (LA_m_), shoot biomass (SM) and root biomass (RM) in late full light (L_FL_), moderate shading (L_MS_) and heavy shading (L_HS_) for *Kmeria septentrionalis* (K), *Celtis sinensis* (C), *Lithocarpus glaber* (L) with early experiences of homogeneous moderate shading (E_hom-MS_), full light (E_hom-FL_) and temporally heterogeneous light (E_het_) conditions. Different letters indicate differences between early experiences for each species within the same late treatments (*P* < 0.05).


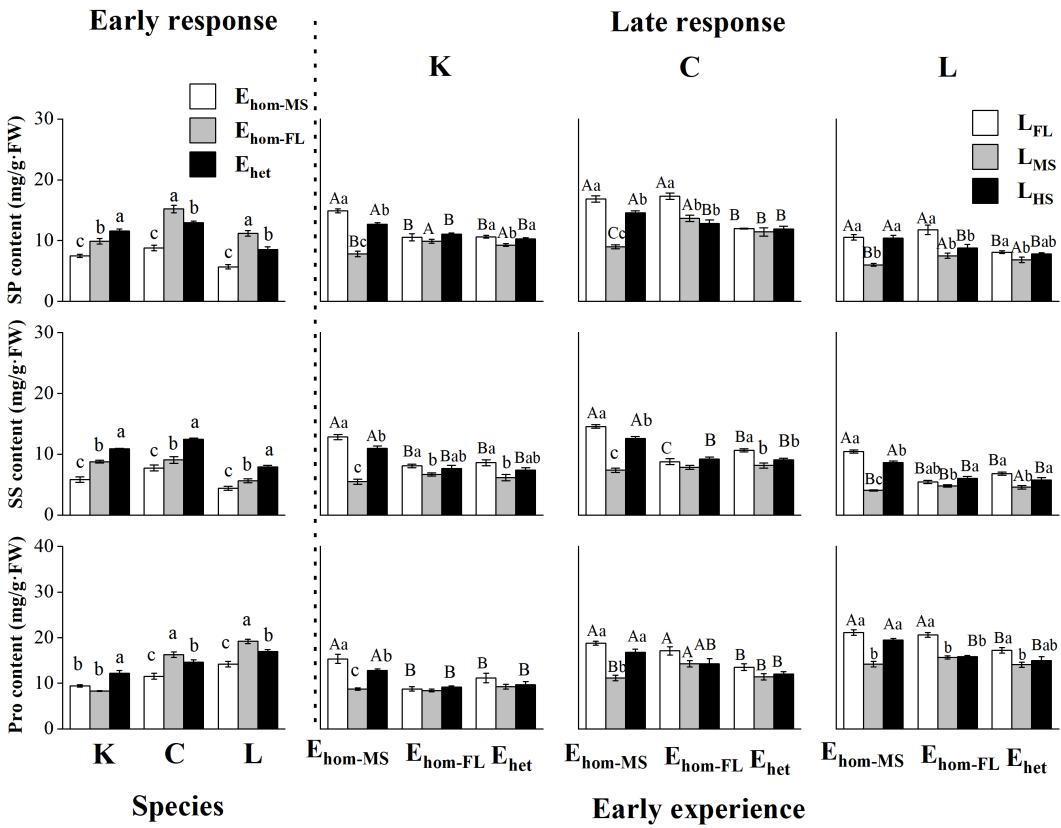
**Fig.S4** Mean contents (±SE) of soluble protein (SP), soluble sugar (SS), free proline (Pro) for *Kmeria septentrionalis* (K), *Celtis sinensis* (C), *Lithocarpus glaber* (L) in homogeneous moderate shading (E_hom-MS_), full light (E_hom-FL_) and temporally heterogeneous light conditions (E_het_) (early experience, in the first round of treatments), and in late full light (L_FL_), moderate shading (L_MS_), heavy shading (L_HS_) after different early experiences (late response, in the second round of treatments). Different lowercase letters indicate differences between early treatments for each species in early response and between late treatments within the same early experiences in late response, different uppercase letters indicate differences between early experiences for each species within the same late treatments in late response (*P* < 0.05).

**
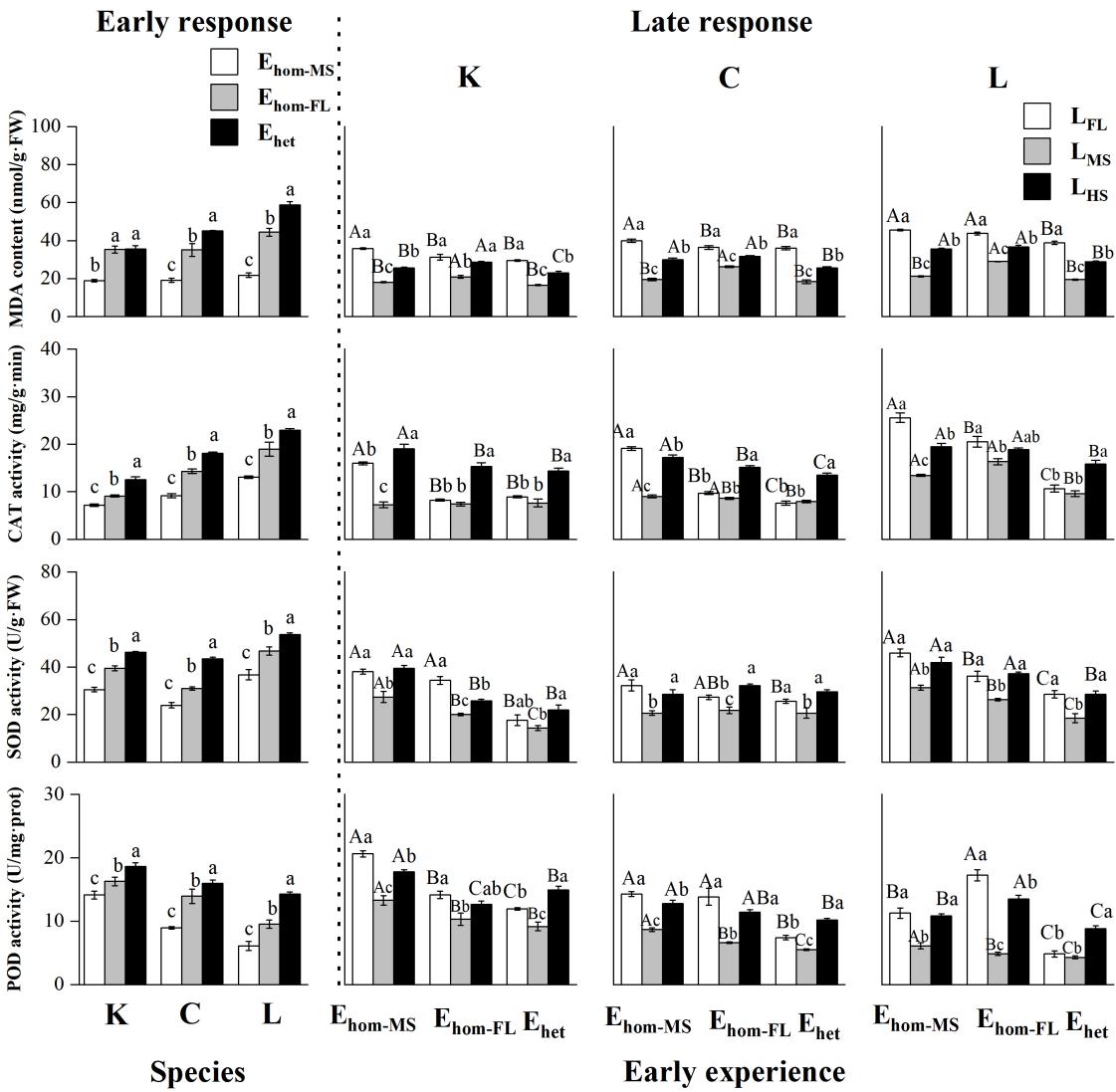
Fig.S5** Mean values (±SE) of malondialdehyde content (MDA), catalase (CAT), superoxide dismutase (SOD) and peroxidase (POD) activity of *Kmeria septentrionalis* (K), *Celtis sinensis* (C), *Lithocarpus glaber* (L) in homogeneous moderate shading (E_hom-MS_), full light (E_hom-FL_) and temporally heterogeneous light conditions (E_het_) (early experience), and in late full light (L_FL_), moderate shading (L_MS_), heavy shading (L_HS_) after different early experiences (late response). Different lowercase letters indicate differences between early treatments for each species in early response and between late treatments within the same early experiences in late response, different uppercase letters indicate differences between early experiences for each species within the same late treatments in late response (*P* < 0.05).

**
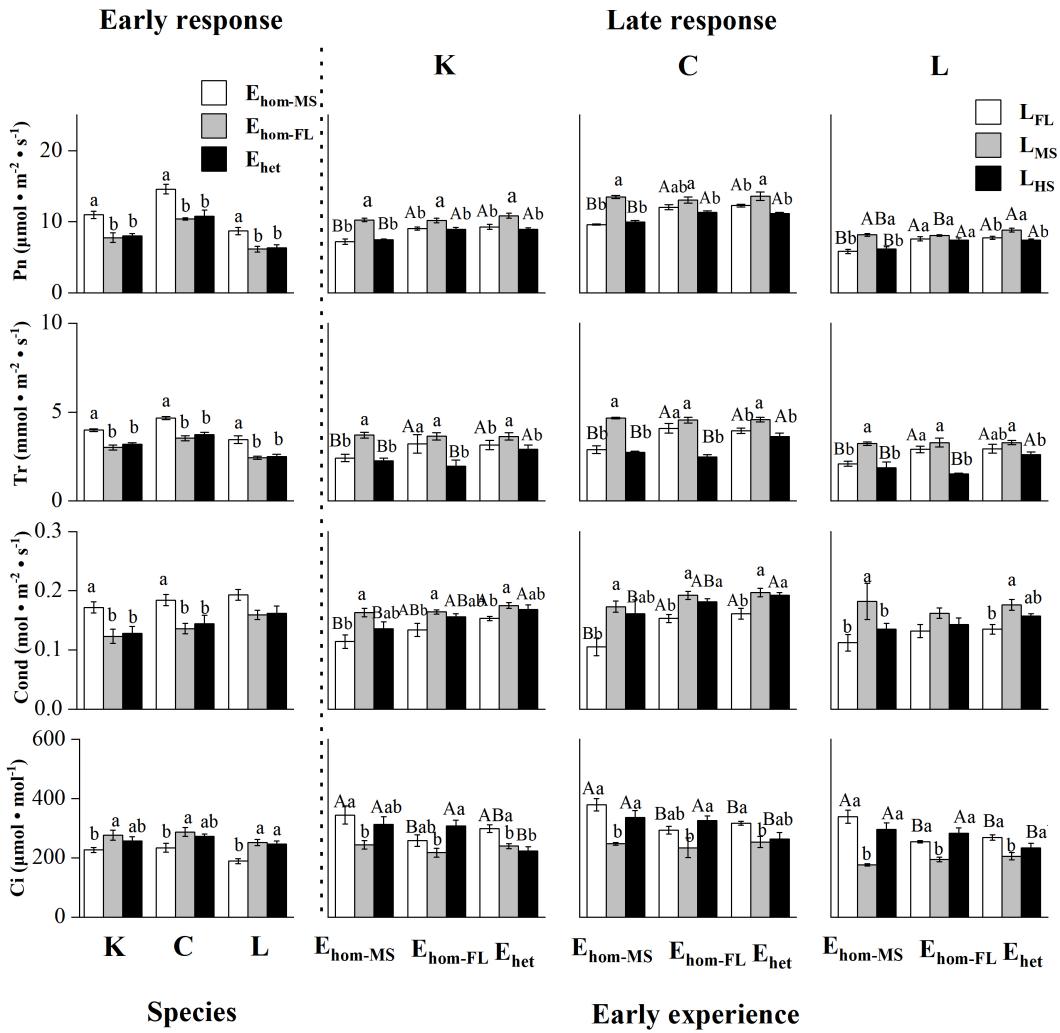
Fig.S6** Mean values (±SE) of net photosynthesis rate (Pn), transpiration rate (Tr), stomatal conductance (Cond), and intercellular CO_2_ concentration (Ci) of *Kmeria septentrionalis* (K), *Celtis sinensis* (C), *Lithocarpus glaber* (L) in homogeneous moderate shading (E_hom-MS_), full light (E_hom-FL_) and temporally heterogeneous light conditions (E_het_) (early experience), and in late full light (L_FL_), moderate shading (L_MS_), heavy shading (L_HS_) after different early experiences (late response). Different lowercase letters indicate differences between early treatments for each species in early response and between late treatments within the same early experiences in late response, different uppercase letters indicate differences between early experiences for each species within the same late treatments in late response (*P* < 0.05).

**Table S1** *F*-values from three-way ANCOVA for effects of species (SP), early treatment (ET), and late treatment (LT) and their interactions on log-transformed stem basal diameter (SD), stem length (SL), maximum leaf area (LA_m_), shoot biomass (SM) and root mass (RM), for plants with two rounds of treatments, with log_10_ (Initial size [IS]) nested in the species as a covariate.

Significance levels: * *P* < 0.05, ** *P* < 0.01, *** *P* < 0.001.

| [Source](http://www.baidu.com/link?url=g5VCm0jTdhSCdxTUZyLBhbJuv1HeUsDbo-UiRgg3psFp4HCQGrQ793PORorJR0uKFZdfXgudQNuWCU2TA7ot9_) | Df | Log_10_ SD | Log_10_ SL | Log_10_ LA_m_ | Log_10_ SM | Log_10_ RM |
| --- | --- | --- | --- | --- | --- | --- |
| Log_10_ (IS) | 1 | 27.03 | 3.48 | 0.05 | 0.02 | 8.05 |
| SP | 2 | **75.75^***^** | **249.01^***^** | **1305.87^***^** | **1291.72^***^** | **2623.46^***^** |
| ET | 2 | **12.08^***^** | **7.78^**^** | **101.78^***^** | **62.46^***^** | **84.94^***^** |
| LT | 2 | 0.20 | 0.68 | **10.86^***^** | 2.66 | **3.36^*^** |
| SP × ET | 4 | 0.46 | 0.64 | **22.17^***^** | **9.40^***^** | **7.11^**^** |
| SP × LT | 4 | 0.05 | 0.03 | 1.42 | 0.22 | 0.06 |
| ET × LT | 4 | 0.28 | 0.27 | 0.40 | 0.52 | 0.91 |
| SP × ET × LT | 8 | 0.16 | 0.03 | 0.11 | 0.06 | 0.09 |

**Table S2** *F*-values from three-way ANOVA for the effects of species (SP), early treatment (ET), and late treatment (LT) and their interactions on late growth in stem basal diameter (LG_SD_), stem length (LG_SL_), maximum leaf area (LG_LAm_), shoot biomass (LG_SM_) and root biomass (LG_RM_), for plants with two rounds of treatments.

| [Source](http://www.baidu.com/link?url=g5VCm0jTdhSCdxTUZyLBhbJuv1HeUsDbo-UiRgg3psFp4HCQGrQ793PORorJR0uKFZdfXgudQNuWCU2TA7ot9_) | Df | LG_SD_ | LG_SL_ | LG_LAm_ | LG_SM_ | LG_RM_ |
| --- | --- | --- | --- | --- | --- | --- |
| SP | 2 | **21.95^***^** | **19.54^***^** | **11.42^***^** | **151.32^***^** | **255.27^***^** |
| ET | 2 | **53.21^***^** | **52.92^***^** | **5.54^**^** | **158.85^***^** | **148.15^***^** |
| LT | 2 | **241.33^***^** | **65.37^***^** | **29.63^***^** | **192.21^***^** | **189.46^***^** |
| SP × ET | 4 | 2.23 | 2.12 | 0.83 | **14.09^***^** | **16.44^***^** |
| SP × LT | 4 | 2.53 | **4.79^*^** | 1.24 | **9.26^**^** | **15.64^***^** |
| ET × LT | 4 | **5.16^**^** | **32.67^***^** | 1.37 | **29.38^***^** | **27.95^***^** |
| SP × ET × LT | 8 | 0.42 | 0.98 | 0.27 | **3.91^*^** | **4.89^*^** |

Significance levels: * *P* < 0.05, ** *P* < 0.01, *** *P* < 0.001.

**Table S3** *F*-values from three-way ANOVA for the effects of species (SP), early treatment (ET), and late treatment (LT) and their interactions on photosynthesis rate (Pn), transpiration rate (Tr), stomatal conductance (Cond), intercellular CO_2_ concentration (Ci), soluble protein (SP), soluble sugar (SS), and free proline (Pro) content, for plants with two rounds of treatments.

| [Source](http://www.baidu.com/link?url=g5VCm0jTdhSCdxTUZyLBhbJuv1HeUsDbo-UiRgg3psFp4HCQGrQ793PORorJR0uKFZdfXgudQNuWCU2TA7ot9_) | Df | Pn | Tr | Coud | Ci | SP | SS | Pro |
| --- | --- | --- | --- | --- | --- | --- | --- | --- |
| SP | 2 | **521.40^***^** | **59.11^***^** | **8.19^***^** | **13.92^***^** | **269.67^***^** | **221.44^***^** | **243.89^***^** |
| ET | 2 | **30.30^***^** | **13.64^***^** | **11.87^***^** | **14.04^***^** | **45.68^***^** | **131.82^***^** | **42.25^***^** |
| LT | 2 | **125.02^***^** | **93.25^***^** | **32.52^***^** | **52.91^***^** | **152.87^***^** | **225.20^***^** | **88.91^***^** |
| SP × ET | 4 | 0.05 | 0.14 | 1.29 | 0.13 | **8.43^***^** | 1.19 | **9.31^***^** |
| SP × LT | 4 | **3.56^*^** | 0.83 | 1.10 | 0.70 | **2.81^*^** | 0.67 | 2.20 |
| ET × LT | 4 | **13.00^***^** | **11.15^***^** | 1.71 | **10.48^***^** | **41.44^***^** | **63.84^***^** | **16.59^***^** |
| SP × ET × LT | 8 | 0.44 | 0.14 | 0.21 | 0.36 | **6.48^**^** | 0.39 | 1.72 |

Significance levels: * *P* < 0.05, ** *P* < 0.01, *** *P* < 0.001.
